# Prognostic Impact of Long-Term Sodium Zirconium Cyclosilicate-Integrated Medical Therapy in Patients with Systolic Heart Failure

**DOI:** 10.3390/jcm14082836

**Published:** 2025-04-20

**Authors:** Yuki Hida, Teruhiko Imamura, Koichiro Kinugawa

**Affiliations:** Second Department of Medicine, University of Toyama, 2630 Sugitani, Toyama 930-0194, Japan

**Keywords:** ke chronic kidney disease, potassium, hyperkalemia

## Abstract

**Background:** Sodium zirconium cyclosilicate (SZC) is a novel potassium-binding agent with strong evidence supporting its efficacy in normalizing hyperkalemia. However, the long-term prognostic impact of SZC-integrated medical therapy in patients with systolic heart failure and baseline hyperkalemia remains uncertain. **Methods:** This study included patients with heart failure and a left ventricular ejection fraction (LVEF) of <50% who were prescribed SZC for hyperkalemia between July 2020 and February 2025. Patients who continued SZC therapy for two years or until February 2025 were classified into the SZC continuation group and followed from the initiation of SZC. Those who discontinued SZC during the study period were assigned to the SZC discontinuation group, with follow-up commencing from the point of cessation. The two-year cumulative incidence of all-cause mortality or hospital readmission was compared between the groups. **Results:** A total of 61 patients (median age: 79 years; 33 men; median LVEF: 42%) were included in the analysis. Serum potassium levels significantly decreased in the SZC continuation group (*p* < 0.001) but remained unchanged in the SZC discontinuation group (*p* = 0.23). The SZC continuation group demonstrated a trend toward a lower cumulative incidence of the primary outcome compared to the SZC discontinuation group (29% vs. 47%, *p* = 0.079). Additionally, in the SZC continuation group, the daily doses of renin-angiotensin system inhibitors and mineralocorticoid receptor antagonists increased significantly (*p* < 0.05 for both). Furthermore, LVEF improved significantly with SZC-integrated medical therapy (*p* = 0.011), whereas no such changes were observed in the SZC discontinuation group (*p* > 0.05 for all). **Conclusions:** Long-term SZC-integrated medical therapy was associated with the sustained normalization of hyperkalemia, optimization of heart failure pharmacotherapy, and improved clinical outcomes in patients with systolic heart failure and baseline hyperkalemia. These findings underscore the need for prospective randomized controlled trials in carefully selected patient populations to validate the benefits of SZC and establish its optimal supportive role in the management of systolic heart failure.

## 1. Introduction

Sodium zirconium cyclosilicate (SZC) is a recently introduced, non-polymer zirconium silicate compound that effectively reduces serum potassium levels by exchanging sodium and hydrogen for potassium and ammonium ions in the gastrointestinal tract [1,2,3]. Robust evidence supports its efficacy in managing hyperkalemia [4,5,6,7,8,9]. During the administration of SZC, serum potassium levels can be maintained within a normal range with few drug-related adverse events.

Emerging data suggest that the concomitant administration of SZC may facilitate the initiation and up-titration of heart failure-specific pharmacotherapies, including renin-angiotensin system (RAS) inhibitors and mineralocorticoid receptor antagonists (MRAs) [10,11,12]. These agents, while fundamental to heart failure management, frequently induce hyperkalemia, thereby limiting their use—particularly in patients with systolic heart failure and chronic kidney disease [13].

The long-term integration of SZC into heart failure management may theoretically enhance clinical outcomes by enabling the maximization and dose optimization of these foundational therapies. However, in real-world clinical practice, many clinicians resort to down-titration of heart failure medications to mitigate hyperkalemia [14,15]. Even when potassium binders, including SZC, are introduced for hyperkalemia management, it is often discontinued shortly after potassium levels normalize [16]. Consequently, the real-world long-term clinical impact of sustained SZC therapy, particularly regarding outcomes such as mortality, remains unclear.

In the present study, we investigated the two-year impact of SZC-integrated medical therapy on mortality and all-cause hospital readmissions in patients with systolic heart failure. Additionally, we examined the association between long-term SZC therapy and changes in heart failure medication dosages, laboratory parameters, and echocardiographic findings.

## 2. Methods

### 2.1. Patient Selection

This study was a retrospective chart review analysis. Patients who received SZC therapy for hyperkalemia between July 2020 and February 2025 were considered eligible for inclusion. Among them, individuals diagnosed with symptomatic systolic heart failure, defined as a left ventricular ejection fraction of <50%, and had an indication of the up-titration of guideline-directed medical therapy were included in the study. Patients who were dependent on hemodialysis or concurrently receiving other potassium-binding agents were excluded.

Informed consent was obtained from all participants prior to their inclusion in the study. Additionally, the study protocol was reviewed and approved by the institutional ethics board in accordance with ethical guidelines.

### 2.2. Study Design

Patients who maintained SZC therapy for two years, until the conclusion of the study period in February 2025, or until their death, were classified into the SZC continuation group. These patients were followed from the initiation of SZC therapy (the time when SZC was initiated was defined as day 0).

Conversely, patients who discontinued SZC at any point during the study period were assigned to the SZC discontinuation group. The follow-up for these patients commenced from the time of SZC cessation (the time when SZC was terminated was defined as day 0) and continued for two years or until the end of the study period, whichever occurred first.

The primary outcome of interest was a composite endpoint of all-cause mortality and hospital readmissions during the observation period. Secondary outcomes included additional clinical parameters, as detailed below. These outcomes were compared between the SZC continuation group and the SZC discontinuation group.

### 2.3. SZC Therapy

SZC therapy was initiated for the management of hyperkalemia, starting with a loading dose administered over two days, in accordance with the institutional protocol. This was followed by a maintenance dose ranging from 5 to 15 g per day, tailored to individual patient needs. The discontinuation of SZC was determined at the discretion of the treating physicians, typically upon the resolution of hyperkalemia.

### 2.4. Heart Failure Medications

Heart failure medications were attempted to up-titrate according to the guidelines to achieve the maximum target dose, in principle [17]. The dose up-titration was conducted at the discretion of the attending cardiologists, dominantly based on patients’ serum potassium levels, renal function, cardiac function, and vital signs. The dose of diuretics was adjusted according to the status of patients’ systemic/pulmonary congestion, electrolyte data, renal function, and vital signs. Vasopressin type 2 receptor antagonist tolvaptan was considered for refractory congestion [17]. The dose of loop diuretics was generally decreased following the initiation of tolvaptan, in principle.

### 2.5. Data Collection

Demographic characteristics, comorbidities, laboratory values, echocardiographic parameters, and medication data were collected at baseline. For the SZC continuation group, baseline was defined as the time of SZC initiation, whereas for the SZC discontinuation group, it was defined as the time of SZC cessation.

Medication dosages were standardized using equivalent dose conversions: beta-blocker doses were expressed as carvedilol equivalents, RAS inhibitor doses as enalapril equivalents, MRA doses as spironolactone equivalents, and loop diuretic doses as furosemide equivalents.

The primary outcome was a composite endpoint of all-cause mortality and hospital readmission events. Serum potassium levels were assessed at baseline and subsequently at 1, 2, 3, 6, and 12 months. Daily doses of heart failure medications were recorded at baseline, as well as at 1-month and 3-month follow-ups. Laboratory data were collected at baseline, 1-month, and 3-month follow-ups, while echocardiographic assessments were performed at baseline and at 3 months.

### 2.6. Statistics

Statistical analyses were performed using SPSS Statistics 23 (SPSS Inc., Armonk, IL, USA). Two-tailed *p*-values < 0.05 were considered statistically significant. Given the small sample size, all variables were treated as non-parametric data. Clinical data were compared between the SZC continuation group and the SZC discontinuation group.

Continuous variables were analyzed using the Mann–Whitney U test, while categorical variables were compared using Fisher’s exact test. Trends in multiple clinical parameters over time were assessed using the Friedman test, and paired comparisons of clinical parameters were conducted using the Wilcoxon signed-rank test. The cumulative incidence of the primary outcome was compared between the two groups using the log-rank test.

Cox proportional hazards regression analysis was performed to identify predictors of the primary outcome, incorporating pre-specified variables, including SZC discontinuation. Variables with *p*-values < 0.20 in univariable analyses were included in the multivariable analysis using a forced-entry method. Additionally, a two-sample Poisson rate comparison was conducted to compare the annual event rates of the all-cause readmissions between the two groups.

Logistic regression analysis was used to identify factors associated with the failure of heart failure medication up-titration within three months. Variables with *p*-values < 0.20 in the univariable analysis, including SZC discontinuation, were included in the multivariable analysis using a forced-entry method.

## 3. Results

### 3.1. Baseline Characteristics

A total of 61 patients were included in this retrospective study (Table 1). The median age was 79 years (interquartile range: 69, 86), and 33 patients (54%) were male. All patients presented with hyperkalemia and were treated with SZC. All had a left ventricular ejection fraction of <50%. The median plasma B-type natriuretic peptide level was 277 pg/mL (176, 461), and the estimated glomerular filtration rate was 28.7 mL/min/1.73 m^2^ (19.0, 41.0). Patients received guideline-directed medical therapy as tolerated, with approximately half also receiving tolvaptan.

Of the total cohort, 19 patients (31%) continued SZC therapy throughout the observation period (SZC continuation group), while 42 patients (69%) discontinued SZC (SZC discontinuation group). Baseline characteristics were assessed at the time of SZC initiation for the SZC continuation group and at the time of SZC cessation for the SZC discontinuation group. No significant differences were observed in baseline characteristics between the two groups, except for serum potassium levels, which were significantly higher in the SZC continuation group than in the SZC discontinuation group (*p* < 0.001).

### 3.2. Trajectory of Serum Potassium Level

**Figure 1:**
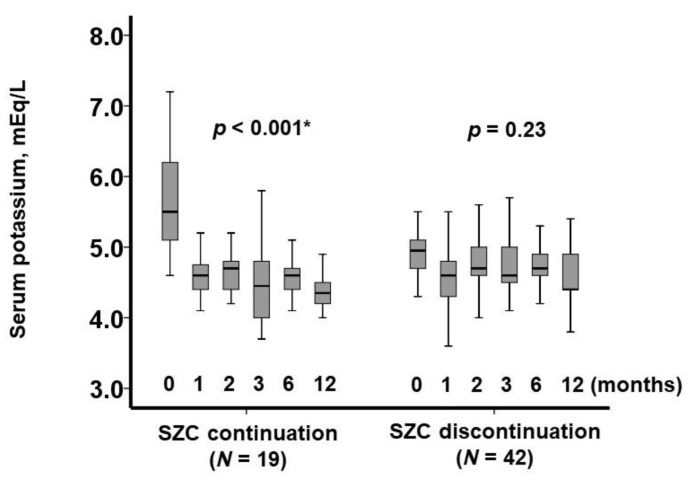
Trajectory of serum potassium levels during one-year observation period. Serum potassium levels are shown for the SZC continuation group following SZC initiation and for the SZC discontinuation group following SZC cessation. * *p* < 0.05 by Friedman test.

In the SZC continuation group, SZC therapy was maintained throughout the observation period, leading to a significant reduction in serum potassium levels over the course of one year (*p* < 0.001; Figure 1). Notably, no patients in this group experienced drug-related adverse events, including hypokalemia or peripheral edema.

In contrast, in the SZC discontinuation group, SZC was withdrawn at day 0. Serum potassium levels exhibited an initial upward trend during the first three months, followed by a gradual decline; however, this change was not statistically significant (*p* = 0.23).

### 3.3. Prognostic Impact of SZC-Incorporated Medical Therapy

Following the initiation of SZC, patients in the SZC continuation group were observed for a median of 730 days (486, 730), while those in the SZC discontinuation group were followed for a median of 654 days (425, 730). During this period, 22 patients (36%) experienced the primary outcome, including 12 (20%) deaths and 18 (30%) hospital readmissions.

**Figure 2:**
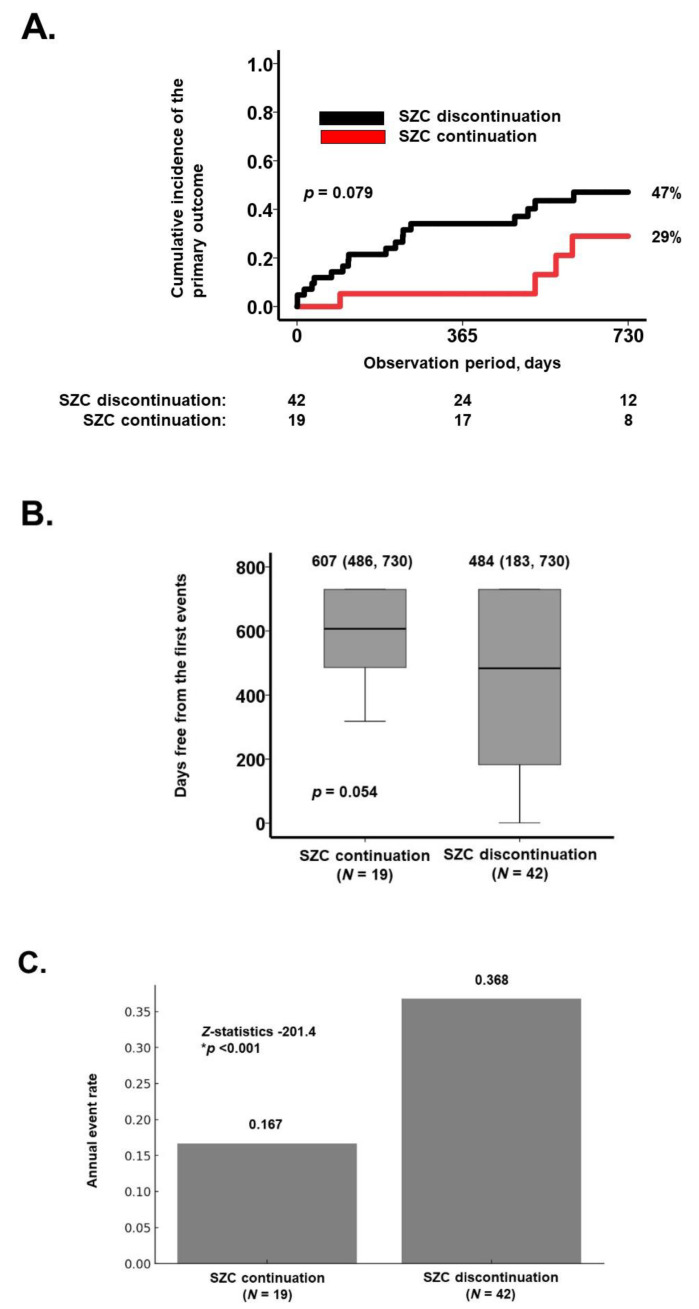
Two-year cumulative incidence of the primary outcome (**A**); days free from the first events (**B**); and the annual event rates (**C**). (**A**) Kaplan–Meier curves comparing the cumulative incidence of the primary outcome between the SZC continuation group and the SZC discontinuation group, analyzed using the log-rank test. (**B**) Comparison of days free from the first event between the two groups using the Mann–Whitney U test. (**C**) Comparison of annual event rates between the two groups using the two-sample Poisson rate comparison test. * *p* < 0.05.

Over the two-year observation period, the cumulative incidence of the primary outcome was lower in the SZC continuation group compared to the SZC discontinuation group (29% vs. 47%), corresponding to an absolute risk reduction of 18% (*p* = 0.079; Figure 2A). Furthermore, the median event-free duration was longer in the SZC continuation group than in the SZC discontinuation group (607 vs. 484 days, *p* = 0.054; Figure 2B). The annual event rate was significantly lower in the SZC continuation group (0.167 vs. 0.368 events per year, *p* < 0.001; Figure 2C).

The prognostic impact of various potential factors on the primary outcome was assessed (Table 2). In the univariable analysis, age, plasma B-type natriuretic peptide level, and SZC discontinuation were identified as potential predictors of the primary outcome (*p* < 0.20). After adjusting for age and plasma B-type natriuretic peptide levels, SZC discontinuation demonstrated a trend toward an association with the primary outcome, with a hazard ratio of 2.48 (95% confidence interval: 0.84–7.37, *p* = 0.082).

### 3.4. Trajectory of Daily Medication Dose

**Figure 3:**
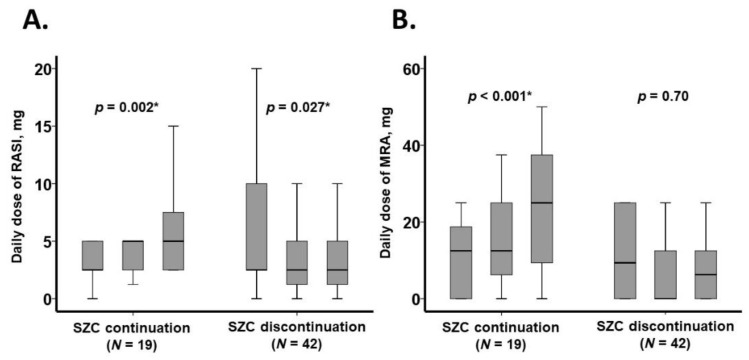
Three-month trajectory of daily dose of heart failure medications: RASI (**A**) and MRA (**B**). Data were collected at baseline, one month, and three months. The daily dose of RASI was expressed in enalapril equivalents, while the daily dose of MRA was expressed in spironolactone equivalents. RASI, renin-angiotensin system inhibitor; MRA, mineralocorticoid receptor antagonist. * *p* < 0.05.

The trajectory of medication doses over the three-month observation period is presented in Figure 3 and Table 3. In the SZC continuation group, the daily doses of RAS inhibitors and MRAs increased significantly (*p* < 0.05 for both). Conversely, in the SZC discontinuation group, the daily dose of RAS inhibitors significantly decreased (*p* = 0.027), while the dose of MRAs remained unchanged (*p* = 0.70) (Figure 3).

The doses of other heart failure medications, including beta-blockers, remained stable regardless of SZC use (*p* > 0.05 for all; Table 2). However, in the SZC discontinuation group, there was a trend toward an increase in the doses of loop diuretics (*p* = 0.076) and tolvaptan (*p* = 0.066).

Failure to up-titrate RAS inhibitors was observed in 32 patients (52%). Among the potential variables analyzed, SZC discontinuation was independently associated with the failure of RAS inhibitor dose escalation, with an adjusted hazard ratio of 20.5 (95% confidence interval: 2.93–144.2, *p* = 0.002), along with an estimated glomerular filtration rate (*p* = 0.043) (Table 4).

Similarly, failure to up-titrate MRAs was observed in 28 patients (46%). Among the potential factors, SZC discontinuation was again independently associated with the failure of MRA dose escalation, with an adjusted hazard ratio of 11.52 (95% confidence interval: 2.57–51.7, *p* = 0.001; Table 5).

### 3.5. Trajectory of Laboratory Data

**Figure 4:**
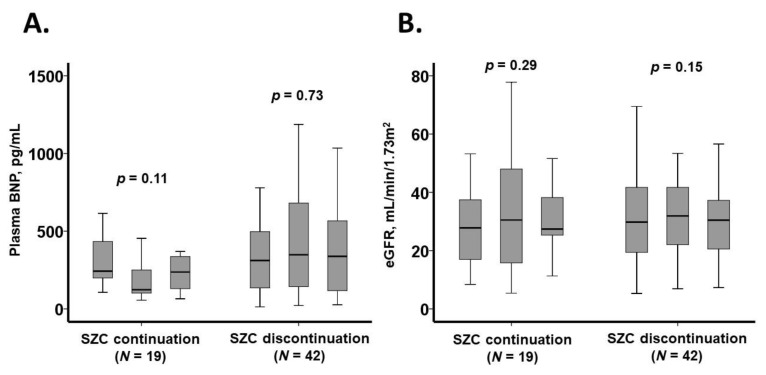
Three-month trajectory of laboratory data: plasma BNP levels (**A**) and eGFR (**B**). Data were collected at baseline, one month, and three months later. BNP, B-type natriuretic peptide; eGFR, estimated glomerular filtration rate.

The trajectory of laboratory data over the three-month observation period is presented in Figure 4 and Table 6. In the SZC continuation group, plasma B-type natriuretic peptide levels showed a trend toward reduction (*p* = 0.11), whereas in the SZC discontinuation group, BNP levels remained unchanged (*p* = 0.73).

The estimated glomerular filtration rate remained stable in both groups, with no significant differences observed regardless of SZC use (*p* > 0.05 for both). Similarly, all other laboratory parameters remained unchanged throughout the observation period, irrespective of SZC therapy (*p* > 0.05 for all).

### 3.6. Trajectory of Echocardiography Data

**Figure 5:**
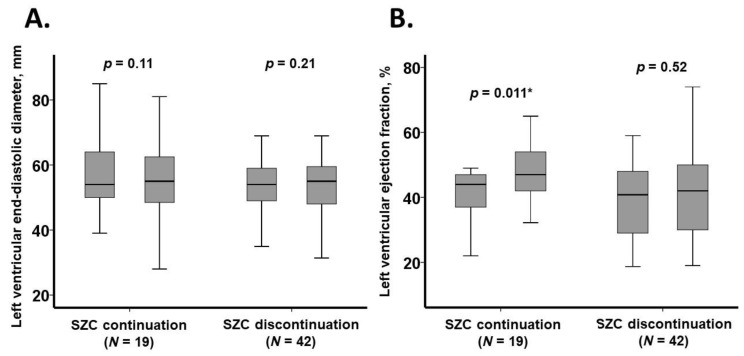
Three-month trajectory of echocardiography data: left ventricular end-diastolic diameter (**A**) and left ventricular ejection fraction (**B**). Data were collected at baseline and three months later. * *p* < 0.05.

Key echocardiographic parameters over the three-month observation period are illustrated in Figure 5. Left ventricular end-diastolic diameter remained unchanged in both the SZC continuation and SZC discontinuation groups (*p* > 0.05 for both). However, left ventricular ejection fraction significantly improved in the SZC continuation group (*p* = 0.011), whereas no significant change was observed in the SZC discontinuation group (*p* = 0.52).

## 4. Discussion

In this retrospective study, we evaluated the long-term impact of SZC-incorporated medical therapy on a composite endpoint of all-cause mortality and hospital readmissions in patients with systolic heart failure and baseline hyperkalemia. Patients who discontinued SZC served as the control group.

In the SZC continuation group, serum potassium levels significantly decreased following SZC initiation. Concurrently, the daily doses of RAS inhibitors and MRAs increased significantly, plasma B-type natriuretic peptide levels exhibited a trend toward reduction, and left ventricular ejection fraction improved significantly.

In the SZC discontinuation group, serum potassium levels remained unchanged throughout the observation period despite the absence of SZC therapy. The daily dose of RAS inhibitors significantly declined, while MRA doses remained unchanged. Similarly, plasma BNP levels and echocardiographic parameters showed no significant changes.

The two-year cumulative incidence of all-cause mortality or hospital readmissions tended to be lower in the SZC continuation group compared to the SZC discontinuation group.

### 4.1. Clinical Impact of SZC Secession

Clinicians traditionally prescribe potassium binders as a temporary intervention to correct hyperkalemia and discontinue them once potassium levels normalize. In the REVEAL-HK trial, approximately 70% of heart failure patients discontinued potassium binders within one year, and nearly 40% experienced recurrent hyperkalemia at the one-year follow-up [16]. This aligns with previous findings from UK primary and secondary care data, where 26% of heart failure patients had hyperkalemia, and 18% experienced recurrent hyperkalemia within six months [18]. Similarly, in the present study, serum potassium levels exhibited an increasing trend during the first few months following SZC discontinuation.

Interestingly, despite the absence of SZC re-administration, serum potassium levels did not continue to rise in the SZC discontinuation group. This phenomenon can be attributed to the down-titration or lack of up-titration of heart failure medications, which are known to elevate serum potassium levels. In other words, clinicians appeared to mitigate the risk of recurrent hyperkalemia by reducing the doses of heart failure medications. Notably, SZC discontinuation emerged as an independent risk factor for the failure of heart failure medication up-titration (see Table 4 and Table 5).

A trend toward increased diuretic use was observed following SZC discontinuation. The reduction in MRA doses, which provide mild diuretic effects [19], may have contributed to decreased urine output, necessitating an up-titration of loop diuretics. The use of high-dose loop diuretics may have unfavorable prognostic implications by activating the renin-angiotensin system and sympathetic nervous system, potentially exacerbating the progression of cardiovascular diseases.

### 4.2. Clinical Implication of SZC Continuation

In the present study, long-term SZC therapy effectively maintained serum potassium levels within the normal range. Notably, this stabilization occurred despite the up-titration of heart failure medications, which are known to elevate serum potassium levels.

These findings align with previous studies demonstrating the benefits of sustained SZC therapy in heart failure management. A multinational study evaluating routine clinical practice across three countries reported that patients receiving SZC were significantly more likely to continue RAS inhibitor therapy at six months compared to those not treated with potassium binders [10]. Furthermore, a randomized controlled trial demonstrated that SZC not only facilitated the maintenance of normokalemia but also enabled the optimization of MRA dosing in patients with systolic heart failure and baseline hyperkalemia [12].

These findings highlight the potential role of SZC in supporting optimal heart failure pharmacotherapy by mitigating the risk of hyperkalemia, thereby allowing for the sustained use of RAS inhibitors and MRAs, which are cornerstone therapies in heart failure management [13].

### 4.3. SZC Continuation and Clinical Outcomes

Current guidelines recommend the use of potassium binders to maintain heart failure medications in patients with systolic heart failure and baseline hyperkalemia [20,21]. However, the long-term clinical impact of SZC-incorporated medical therapy remains unclear.

In the present study, SZC continuation was associated with a gradual decline in plasma B-type natriuretic peptide levels and stable renal function, likely facilitated by the up-titration of heart failure medications. Importantly, no patients experienced SZC-related gastrointestinal adverse events, which are commonly reported with other potassium binders [7].

Few studies have specifically examined the prognostic impact of SZC-integrated therapy. In the REALIZE-K trial, although limited by statistical power, heart failure recurrence was observed more frequently in patients receiving SZC compared to those on placebo [12]. In a separate study evaluating extremely elderly patients following transcatheter aortic valve replacement, MRA administration was paradoxically associated with an increased incidence of death or heart failure readmission, alongside worsening renal function [22]. In contrast, our findings suggest that long-term SZC therapy may have a beneficial impact on clinical outcomes, as the two-year cumulative incidence of all-cause mortality or hospital readmissions tended to be lower in the SZC continuation group. While SZC enables the up-titration of heart failure medications by mitigating hyperkalemia, further research is necessary to refine patient selection criteria to maximize the clinical benefits of SZC-facilitated heart failure pharmacotherapy.

### 4.4. Limitations

This study serves as a proof-of-concept investigation with a relatively small sample size. When the alpha value is 0.05 and the beta-value is 0.80, the estimated sample size to achieve statistical significance was calculated as 113 versus 113. Although we followed the patients for two years at maximum, we collected and analyzed comprehensive clinical data obtained within three months. Several non-significant differences observed in the two-group comparison may have reached statistical significance in larger-scale studies. Clinical data may not persist during longer observation periods due to medication change or disease progression. Our findings require validation through multi-institutional, large-scale randomized controlled trials [23].

Given the retrospective nature of this study, the influence of potential confounders cannot be entirely eliminated. Although we adjusted for key clinical variables, inherent biases remain a limitation, such as poorer adherence and more advanced diseases in the SZC discontinuation group. Given the small event numbers, we could not include many variables in the multivariable models. We cannot exclude the survivor bias in the SZC discontinuation group. Furthermore, the control group (SZC discontinuation group) in the present study did not account for the placebo effect. Further prospective randomized controlled trials are necessary to confirm our findings. However, in the contemporary clinical landscape, the immediate discontinuation of SZC following hyperkalemia resolution or no therapeutic intervention to persistent hyperkalemia may not be ethically justifiable in future prospective studies, as it could compromise the long-term management of heart failure medications [17].

In the present study, day 0 in the SZC discontinuation group was set at the time when SZC was terminated. They were not affected by hyperkalemia at the actual beginning of the study. An alternative study design may be to set day 0 for the SZC discontinuation group at the time when hyperkalemia developed again following the termination of SZC. However, in real-world clinical practice, clinicians sometimes down-titrate the dose of heart failure medications before recurrent hyperkalemia following the termination of SZC, and recurrent hyperkalemia may not necessarily be encountered.

Additionally, in patients with more advanced heart failure, the presence of cardio-renal syndrome may exacerbate hypotension and renal dysfunction—major barriers to optimizing heart failure therapy, in addition to hyperkalemia [24]. As a result, the applicability of our findings to patients with more severe heart failure with multiple comorbidities remains uncertain and warrants further investigation.

### 4.5. Conclusions

Long-term SZC-integrated medical therapy was associated with the up-titration of RAS inhibitors and MRAs, improved cardiac loading conditions, facilitation of reverse remodeling, and a relatively lower incidence of all-cause mortality or hospital readmissions in patients with systolic heart failure and baseline hyperkalemia. Further studies are needed to refine patient selection criteria and establish the optimal clinical application of long-term SZC-integrated therapy in heart failure management.

## Figures and Tables

**Table 1:** Baseline characteristics.

	Total (*N* = 61)	SZC Continuation (*N* = 19)	SZC Discontinuation (*N* = 42)	*p*-Value
Demographics				
Age, years	79 (69, 86)	78 (66, 84)	79 (71, 87)	0.56
Male	33 (54%)	10 (53%)	23 (55%)	0.55
Body mass index, kg/m^2^	21.5 (19.8, 24.2)	21.5 (20.2, 25.0)	21.7 (19.5, 23.9)	0.55
Comorbidity				
Diabetes mellitus	27 (44%)	8 (42%)	19 (45%)	0.52
Atrial fibrillation	18 (31%)	7 (37%)	11 (26%)	0.29
Coronary artery disease	32 (52%)	11 (58%)	21 (50%)	0.39
Laboratory data				
Hemoglobin, g/dL	11.3 (9.8, 12.2)	11.6 (9.9, 12.4)	11.1 (9.8, 12.1)	0.65
Serum albumin, g/dL	3.3 (2.9, 4.0)	3.7 (3.0, 4.0)	3.2 (2.9, 4.0)	0.38
Serum sodium, mEq/L	138 (136, 141)	138 (136, 141)	138 (136, 141)	0.85
Serum potassium, mEq/L	5.5 (4.7, 6.1)	5.5 (5.1, 6.2)	5.1 (4.7, 6.0)	0.15
Plasma BNP, pg/mL	277 (176, 461)	243 (199, 434)	312 (145, 480)	0.83
eGFR, mL/min/1.73 m^2^	28.7 (19.0, 41.0)	27.8 (17.0, 37.5)	29.3 (19.2, 41.5)	0.75
Echocardiography data				
LVDD, mm	54 (50, 61)	54 (50, 64)	54 (49, 60)	0.73
LVEF, %	42 (32, 48)	44 (37, 47)	41 (28, 48)	0.20
Left atrial diameter, mm	42 (38, 46)	42 (40, 49)	41 (37, 45)	0.43
MR none/trace/mild	36/19/6	10/6/3	26/13/3	0.55
TR none/trace/mild	46/14/1	12/7/0	1934/7/1	0.19
Medication				
Beta-blockers	53 (87%)	18 (95%)	35 (83%)	0.22
Beta-blocker dose, mg/day	5.0 (2.5, 10)	10 (5, 12.5)	5 (2.5, 10)	0.091
RAS inhibitors	60 (98%)	19 (100%)	41 (98%)	0.50
RAS inhibitor dose, mg/day	2.5 (2.5, 5.0)	2.5 (2.5, 5.0)	2.5 (2.5, 7.5)	0.62
MRA	33 (54%)	10 (53%)	23 (55%)	0.55
MRA dose, mg/day	6.25 (0, 12.5)	12.5 (0, 25)	3.75 (0, 12.5)	0.95
SGLT2 inhibitors	25 (41%)	10 (53%)	15 (36%)	0.17
Loop diuretics	41 (67%)	12 (63%)	29 (69%)	0.43
Loop diuretic dose, mg/day	20 (0, 20)	20 (0, 20)	20 (0, 20)	0.87
Tolvaptan	27 (44%)	5 (26%)	22 (52%)	0.058
Tolvaptan dose, mg/day	0 (0, 7.5)	0 (0, 3.75)	0 (3.75, 7.5)	0.11

BNP, B-type natriuretic peptide; eGFR, estimated glomerular filtration rate; LVDD, left ventricular end-diastolic diameter; LVEF, left ventricular ejection fraction; MR, mitral regurgitation; TR, tricuspid regurgitation; RAS, renin-angiotensin system; MRA, mineralocorticoid receptor antagonist; SGLT2, sodium-glucose cotransporter 2. Continuous variables were stated as medina (25% interquartile, 75% interquartile) and compared between the two groups by Mann–Whitney U test. Categorical variables were stated as numbers (percentages) and compared between the two groups using Fischer’s exact test.

**Table 2:** Potential factors associated with the primary outcome.

	Univariable Analysis		Multivariable Analysis	
	Hazard Ratio (95% CI)	*p*-Value	Hazard Ratio (95% CI)	*p*-Value
Age, years	0.98 (0.96–1.01)	0.17	0.98 (0.96–1.01)	0.15
Atrial fibrillation	1.45 (0.61–3.47)	0.40		
Hemoglobin, g/dL	1.04 (0.76–1.30)	0.76		
eGFR, mL/min/1.73 m^2^	1.00 (0.97–1.03)	0.98		
Plasma BNP, pg/mL	1.00 (0.99–1.01)	0.15	1.00 (0.99–1.01)	0.15
SZC discontinuation	2.55 (0.86–7.55)	0.091	2.48 (0.84–7.37)	0.082

CI, confidence interval; eGFR, estimated glomerular filtration rate; BNP, B-type natriuretic peptide. Potential variables were included in the univariable Cox proportional hazard ratio regression analysis to evaluate their impacts on the primary outcome. Variables with *p* < 0.20 in the univariable analyses were included with a forced method in the multivariable analysis.

**Table 3:** Trajectory of medication doses.

	Baseline	1 month	3 months	*p*-Value
SZC continuation (*N* = 19)				
Beta-blocker dose, mg/day	10 (5, 15)	10 (5, 15)	10 (3.75, 15)	0.72
Loop diuretics dose, mg/day	15 (0, 20)	10 (0, 20)	10 (0, 20)	0.28
Tolvaptan dose, mg/day	0 (0, 3.75)	0 (0, 3.75)	0 (0, 3.75)	0.37
SZC discontinuation (*N* = 42)				
Beta-blocker dose, mg/day	10 (2.5, 10)	7.5 (5.0, 10)	10 (3.75, 10)	0.41
Loop diuretics dose, mg/day	10 (0, 20)	20 (0, 20)	30 (0, 30)	0.076
Tolvaptan dose, mg/day	0 (0, 3.75)	0 (0, 7.5)	3.75 (0, 7.5)	0.066

Trajectory of medication dose at baseline, one month, and three months during the observation period was displayed. Data on the SZC continue group and those on the SZC discontinuation group were displayed separately. Continuous variables were stated as medina (25% interquartile, 75% interquartile). The trend was assessed using Friedman test.

**Table 4:** Factors associated with failure of up-titration of RAS inhibitor doses.

	Univariable Analysis		Multivariable Analysis	
	Hazard Ratio (95% CI)	*p*-Value	Hazard Ratio (95% CI)	*p*-Value
Age, years	0.99 (0.94–1.04)	0.67		
Hemoglobin, g/dL	2.64 (0.70–1.42)	0.65		
eGFR, mL/min/1.73 m^2^	0.96 (0.92–1.01)	0.084	0.94 (0.89–0.99)	0.043 *
Plasma BNP, pg/mL	1.00 (0.99–1.01)	0.16	1.01 (0.99–1.01)	0.30
LVEF, %	0.97 (0.90–1.04)	0.37		
Daily dose of MRAs	0.98 (0.92–1.04)	0.44		
SZC discontinuation	11.9 (2.56–55.5)	0.002 *	20.5 (2.93–144.2)	0.002 *

CI, confidence interval; eGFR, estimated glomerular filtration rate; BNP, B-type natriuretic peptide; LVEF, left ventricular ejection fraction; MRA, mineralocorticoid receptor antagonist. Potential variables were included in the univariable logistic regression analyses to evaluate their impacts on the failure of up-titration of MRA doses. Variables with *p* < 0.20 in the univariable analyses were included with a forced method in the multivariable analysis. * *p* < 0.05.

**Table 5:** Factors associated with failure of up-titration of MRA doses.

	Univariable Analysis		Multivariable Analysis	
	Hazard Ratio (95% CI)	*p*-Value	Hazard Ratio (95% CI)	*p*-Value
Age, years	0.96 (0.90–1.01)	0.10	0.95 (0.88–1.02)	0.14
Hemoglobin, g/dL	0.94 (0.68–1.31)	0.72		
eGFR, mL/min/1.73 m^2^	0.98 (0.95–1.02)	0.34		
Plasma BNP, pg/mL	1.00 (0.99–1.01)	0.40		
LVEF, %	0.99 (0.93–1.06)	0.80		
Daily dose of RAS inhibitors	1.01 (0.95–1.07)	0.68		
SZC discontinuation	11.04 (2.66–45.8)	0.001 *	11.52 (2.57–51.7)	0.001 *

CI, confidence interval; eGFR, estimated glomerular filtration rate; BNP, B-type natriuretic peptide; LVEF, left ventricular ejection fraction; RAS, renin-angiotensin system. Potential variables were included in the univariable logistic regression analyses to evaluate their impacts on the failure of up-titration of MRA doses. Variables with *p* < 0.20 in the univariable analyses were included with a forced method in the multivariable analysis. * *p* < 0.05.

**Table 6:** Trajectory of laboratory data.

	Baseline	1 month	3 months	*p*-Value
SZC continuation (*N* = 19)				
Hemoglobin, g/dL	3.9 (3.6, 4.1)	3.6 (3.5, 4.1)	3.7 (3.5, 4.1)	0.86
Serum albumin, g/dL	11.7 (11.0, 12.2)	11.3 (11.1, 11.7)	11.3 (10.7, 11.8)	0.20
Serum sodium, mEq/L	139 (136, 141)	140 (139, 141)	140 (138, 141)	0.17
SZC discontinuation (*N* = 42)				
Hemoglobin, g/dL	11.7 (10.6, 13.0)	11.19 (10.6, 12.4)	11.1 (10.4, 12.8)	0.35
Serum albumin, g/dL	3.5 (3.1, 4.1)	3.6 (3.2, 4.0)	3.7 (3.3, 4.0)	0.34
Serum sodium, mEq/L	139 (137, 141)	140 (138, 142)	140 (138, 141)	0.70

Trajectory of laboratory data at baseline, one month, and three months during the observation period was displayed. Data on the SZC continuation group and those on the SZC discontinuation group were displayed separately. Continuous variables were stated as medina (25% interquartile, 75% interquartile). The trend was assessed using Friedman test.

## Data Availability

The original contributions presented in this study are included in the article. Further inquiries can be directed to the corresponding author(s).
